# Structure and Population of Complex Ionic Species in FeCl_2_ Aqueous Solution by X-ray Absorption Spectroscopy

**DOI:** 10.3390/molecules27030642

**Published:** 2022-01-19

**Authors:** Uroš Luin, Iztok Arčon, Matjaz Valant

**Affiliations:** 1 Materials Research Laboratory, University of Nova Gorica, Vipavska 13, SI-5001 Nova Gorica, Slovenia; uros.luin@ung.si (U.L.); iztok.arcon@ung.si (I.A.); 2 Department of Low and Medium Energy Physics, J. Stefan Institute, Jamova 39, SI-1001 Ljubljana, Slovenia

**Keywords:** structure, population, ionic species, aqueous ferrous chloride, in situ X-ray absorption spectroscopy

## Abstract

Technologies for mass production require cheap and abundant materials such as ferrous chloride (FeCl_2_). The literature survey shows the lack of experimental studies to validate theoretical conclusions related to the population of ionic Fe-species in the aqueous FeCl_2_ solution. Here, we present an in situ X-ray absorption study of the structure of the ionic species in the FeCl_2_ aqueous solution at different concentrations (1–4 molL^−1^) and temperatures (25–80 °C). We found that at low temperature and low FeCl_2_ concentration, the octahedral first coordination sphere around Fe is occupied by one Cl ion at a distance of 2.33 (±0.02) Å and five water molecules at a distance of 2.095 (±0.005) Å. The structure of the ionic complex gradually changes with an increase in temperature and/or concentration. The apical water molecule is substituted by a chlorine ion to yield a neutral Fe[Cl_2_(H_2_O)_4_]^0^. The observed substitutional mechanism is facilitated by the presence of the intramolecular hydrogen bonds as well as entropic reasons. The transition from the single charged Fe[Cl(H_2_O)_5_]^+^ to the neutral Fe[Cl_2_(H_2_O)_4_]^0^ causes a significant drop in the solution conductivity, which well correlates with the existing conductivity models.

## 1. Introduction

Understanding electrolyte solution properties, such as a population of ionic species, local structure, coordination, hydration, and ion association as a function of concentration and temperature, is important for numerous emerging applications, especially for the growing field of energy storage [1,2] The development of new technologies for mass production relies on cheap and abundant materials [3,4,5]. This supports the use of iron in the form of ferrous chloride for different applications. In the field of energy storage application, it is used as an aqueous electrolyte for iron flow batteries [3,6,7,8,9,10], and Power-to-Solid energy storage technology [11]. Ferrous chloride is used in a manufacturing process of the lithium iron orthosilicate (Li_2_FeSiO_4_) [12] and FeSe_2_ cathode [13], FeCl_2_-graphite composite anode used for lithium-ion batteries [14], and Na-FeCl_2_ batteries [15]. Additionally, the use of ferrous chloride is reported for wearable thermoelectric cells [16,17], in environmental applications for arsenic stabilization [18], as well in spintronics for the growth of single-layer magnetic material [19,20].

The existing commercial flow battery technologies have limited energy density due to the solubility limits of the electroactive species [21]. The energy losses of the electrochemical cell are caused by electrodes resistance, electrolyte resistance, and the steric impediment of the ion exchange membranes [22,23,24,25]. For polymeric separators, such as ion exchange membranes used for electrodialysis, reverse osmosis, flow batteries, etc., there is required a fundamental understanding of the influence of ion size and ion charge on salt transport properties [26]. Consequently, the optimization of electrodes, electrolytes, membranes, and other crucial parts of the electrochemical devices is recognized as the important key requirement for the future deployment of new energy storage systems [27].

Molecular dynamic simulations [28,29,30] and experimental analyses [31,32,33] showed that the first hydrated shell of Fe^2+^ in a pure aqueous solution is characterized by a regular octahedron with a Fe-O distance of about 2.10 Å. A density functional theory analysis of the optimized structures predicted a Fe(H_2_O)_6_^2+^ octahedral coordination with inequivalent Fe-O distances suggesting the Jahn–Teller distortion [34]. Opposite to that, four studies ruled out the occurrence of the Jahn–Teller distortion in an Fe^2+^ aqueous solution [31,33,35,36]. The first one was done by combined ab initio quantum mechanical/molecular mechanical molecular dynamics simulations [35], while the other studies involved both the molecular dynamics simulations and X-ray absorption structure method [31,33,36]. In the second hydration shell, 12.4 water molecules were found [35]. 

In the chloride solutions, the chloride ions participate in the structure of Fe ionic species. Lee et al. [37] calculated, based on estimated equilibrium constants, that in the FeCl_2_ solution, the predominant ionic species are [FeCl]^+^ ions. In the entire concentration range, their share does not drop below 90%. According to them, the other two significantly represented species are Fe^2+^ and [FeCl_2_]^0^. With the increase in FeCl_2_ concentration, the share of Fe^2+^ decreases on account of an increase in the neutral [FeCl_2_]^0^. This was confirmed for high temperatures (>200 °C), high pressures (>300 bar), and high Cl concentrations [38,39]. The calculated ion association constant for the association of Fe^2+^ and Cl^−^ to FeCl^+^ is −0.88(5) [40].

Zhao et al. [41] calculated the stability constant of different Fe-chloride complexes from UV and near IR spectra. The results are not consistent with that of Lee et al. [37]. Their calculations showed that Fe^2+^ ions are predominantly present in highly concentrated FeCl_2_ solutions (up to about 3 molL^−1^). Only then did the other complex Fe-chlorine species start to prevail. They also claim that with an increase in concentration and temperature, the Fe-complexes undergo a configurational transformation from octahedral to tetrahedral coordination. The transformation is driven by an increase in entropy change due to a replacement of water molecules by the chloride ions [41].

The literature survey shows that the experimental confirmation of the theoretical studies, related to the population of different ionic Fe-species in ferrous chloride (FeCl_2_) aqueous solution (hereafter FeCl_2_ (aq)), have not been performed. This is critical because the theoretical studies have not offered to same conclusions and, consequently, we cannot be sure what real ionic structures in this solution are. For that reason, we focused on the experimental investigation of ionic species present in the FeCl_2_ (aq) at different concentrations and temperatures. X-ray absorption spectroscopy (XAS) is the method of choice to study the valence state and local structure of the ionic species in solutions and disordered materials [42,43,44,45,46]. We used in situ Fe K-edge XANES (X-ray Absorption Near Edge Structure) and EXAFS (Extended X-ray Absorption Fine Structure) analysis to monitor the Fe valence state [32,45,47] and local structure around the Fe cations in the aqueous solutions [31,32,33]. The target concentration range was from 1 to 4 molL^−1^ and the temperature range was from 25 °C to 80 °C. Using the conductivity model developed by Zhang et al. [48], we showed that the conclusions of the XAS studies well correlate with the conductivity measurements.

## 2. Results

### 2.1. Fe K-Edge XANES Results

**Figure 1:**
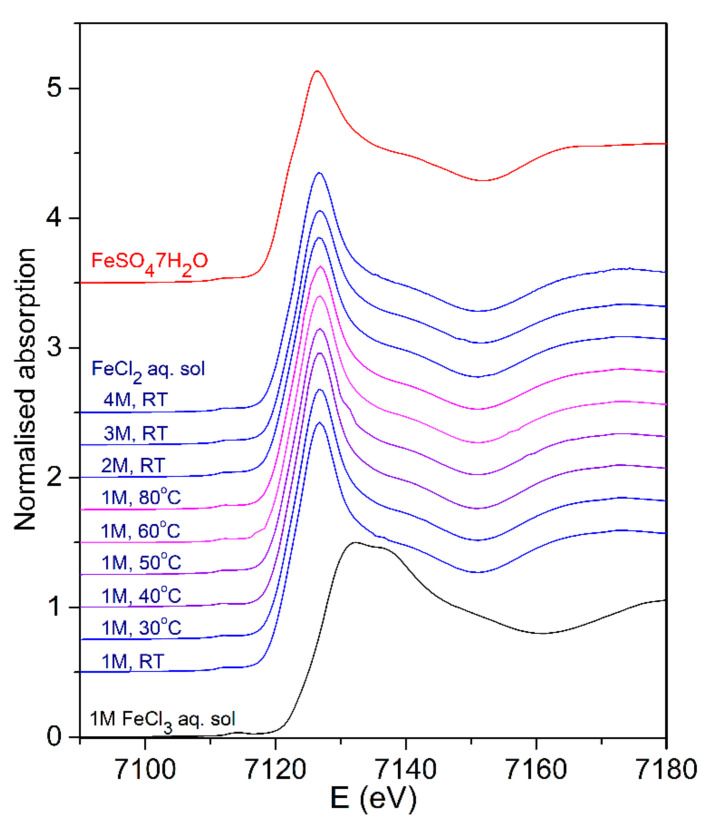
Normalized Fe K-edge XANES spectra of 1 molL^−1^ FeCl_2_ (aq) measured in situ from RT to 80 °C, on FeCl_2_ (aq) at higher concentrations (2, 3, and 4 molL^−1^) at RT, and on 1 molL^−1^ FeCl_3_ (aq) as a reference for Fe^3+^, and crystalline FeSO_4_·7H_2_O as a reference for octahedrally coordinated Fe^2+^ cations.

The Fe K-edge XANES analysis is used to determine the valence state and local symmetry of Fe cations in the 1 molL^−1^ FeCl_2_ (aq) in a temperature range from RT to 80 °C and in the FeCl_2_ (aq) with higher concentrations up to 4 molL^−1^. Normalized Fe K-edge XANES spectra are shown in Figure 1, together with the spectra of selected Fe reference compounds with known Fe valence states and local symmetry of Fe atom neighborhood (1 molL^−1^ FeCl_3_ (aq) as a reference for Fe^3+^ and crystalline FeSO_4_·7H_2_O as a reference for octahedrally coordinated Fe^2+^ cations).

Different local environments of the Fe cations result in different Fe K-edge profiles and pre-edge lines in the XANES spectra. The energy position of the Fe absorption edge and the pre-edge features are correlated with the valence state of the absorbing atom in the sample. With increasing oxidation, each absorption feature in the XANES spectrum is shifted to higher energies. The Fe K-edge shift of about 4.5 eV was found between the spectra of the two- and three-valent iron compounds [32,45,47,49].

The Fe K-edge XANES spectra of all FeCl_2_ (aq) (Figure 1) exhibit the same edge profile, characteristic for octahedrally coordinated Fe^2+^ cations, as in the case of reference FeSO_4_·7H_2_O sample or [Fe(H_2_O)_6_]^2+^ complexes in water solution [32]. The energy position of the Fe K-edge in all FeCl_2_ (aq) solutions is identical, coinciding with the edge position of the reference FeSO_4_·7H_2_O compound. Therefore, the XANES results show that all Fe cations in the FeCl_2_ (aq) at all investigated concentrations up to 4 molL^−1^ are in divalent form with octahedral coordination in the nearest coordination shell. There are also no changes of Fe oxidation state in the solution during heating up to 80 °C. The results give no indications for the presence of tetrahedral Fe(II)-chloride complex [FeCl_4_] with a characteristic pre-edge peak in XANES spectrum at 7112 eV [33,49], which is in agreement with previous findings that indicated the formation of tetrahedral FeCl_4_ species in the aqueous solution at RT only at high Cl concentrations above 8 molL^−1^ or at high temperatures above 150 °C at 6 molL^−1^ [33,49].

### 2.2. Fe K-Edge EXAFS Results

**Figure 2:**
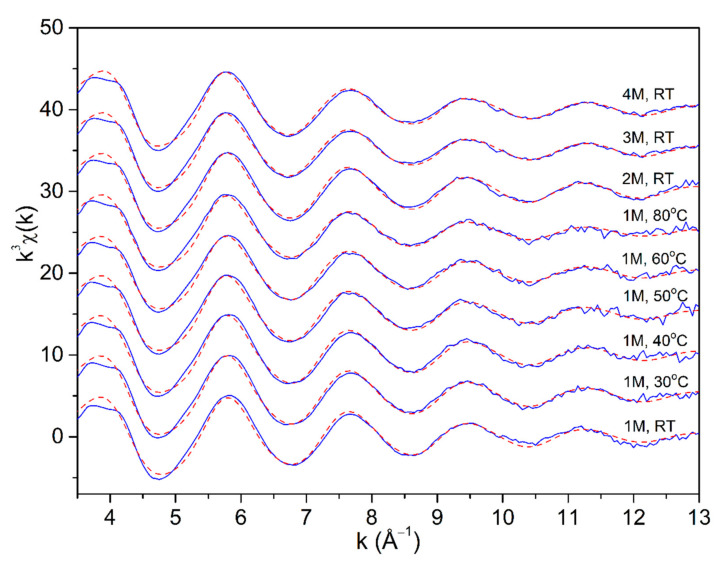
The *k*^3^—weighted Fe K-edge EXAFS spectra of the 1 molL^−1^ FeCl_2_ (aq) in the temperature range from RT to 80 °C, and in the FeCl_2_ (aq) at higher concentrations (2 molL^−1^, 3 molL^−1^, and 4 molL^−1^) at RT, (blue solid line—experiment, red dashed line—best fit EXAFS model in the R range of 1–5 Å). The spectra are displaced vertically for clarity.

**Figure 3:**
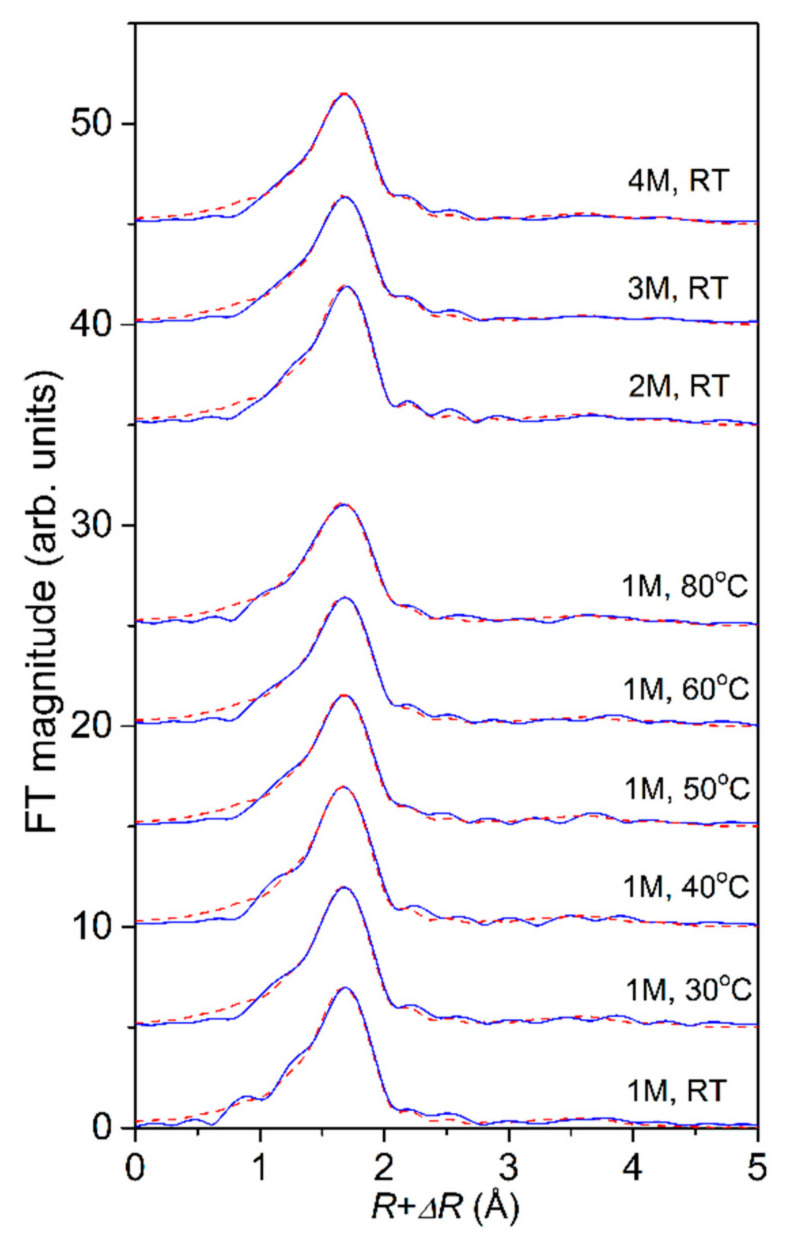
Fourier transform magnitudes of the *k*^3^—weighted Fe K-edge EXAFS spectra of the 1 molL^−1^ FeCl_2_ (aq) in the temperature range from RT to 80 °C, and in the FeCl_2_ (aq) at higher concentrations (2 molL^−1^, 3 molL^−1^, and 4 molL^−1^) at RT, calculated in the *k* range of 3.4 Å^−1^ to 13 Å^−1^ (blue solid line—experiment, red dashed line—best fit EXAFS model in the R range of 1–5 Å).

**Figure 4:**
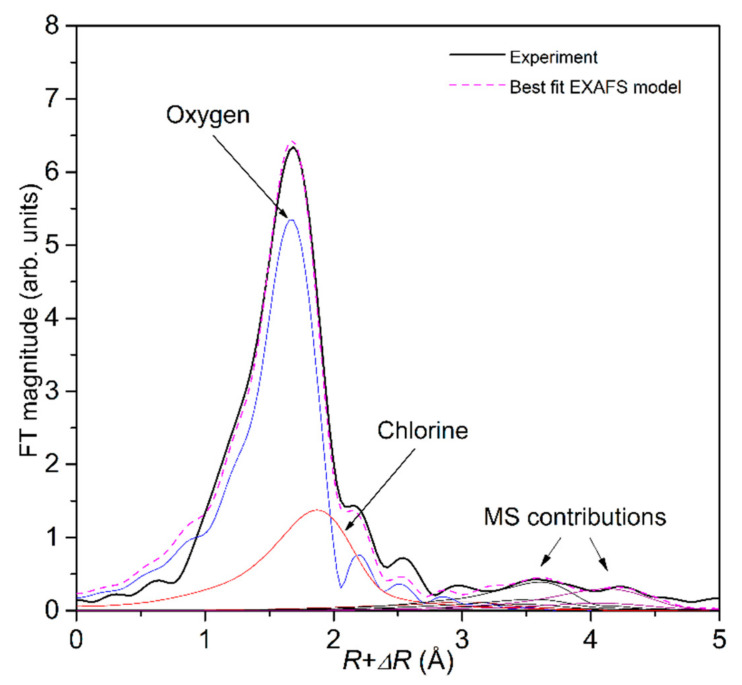
Fourier transform magnitude of *k*^3^—weighted Fe EXAFS spectrum of the 3 molL^−1^ FeCl_2_ (aq) measured at RT, calculated in the *k* range of 3.4–13 Å^−1^. Experiment (black solid line), best-fit EXAFS model in the R range of 1–5 Å (magenta dashed line), and single scattering contribution of the Fe-O (blue line) and Fe-Cl neighbors (red line), and contributions of multiple scatterings with a total path length up to 5 Å.

The Fe K-edge EXAFS analysis was used to directly probe the local structure around Fe cations in the selected temperature and concentration range. Fourier transforms (FT) of the k^3^-weighted Fe K-edge EXAFS spectra (Figure 2, Figure 3 and Figure 4) exhibit a strong peak at about 2 Å, which can be ascribed to photoelectron backscattering on neighbor atoms in the first Fe coordination shell, while the signal from second and more distant Fe hydration shells in the solution is negligible. The weak signal in the R range between 3 and 5 Å can be attributed to the multiple scatterings of the photoelectron within the nearest coordination shell of neighbors. Qualitative comparisons of the Fe FT EXAFS spectra in the R range between 1 and 2.5 Å show that the average Fe neighborhood in the solutions is very similar but not the same. A progressive decrease of the nearest neighbor peak is observed with an increase in temperature and concentration of the FeCl_2_ (aq).

**Figure 5:**
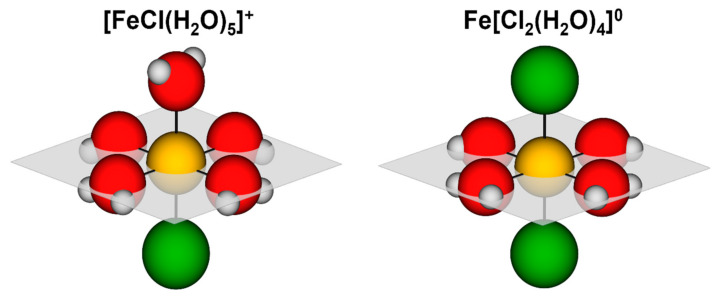
Schematic presentation of two major Fe-ionic species in FeCl_2_ (aq). Fe^2+^ (yellow) in the monovalent [FeCl(H_2_O)_5_]^+^ is coordinated by five water molecules (red and gray) and an apical Cl^−^ ion (green). The intramolecular hydrogen bond restrains movements of the basal water molecules and destabilizes the apical water molecule. The neutral Fe[Cl_2_(H_2_O)_4_]^0^ is formed by substitution of the apical water molecule by Cl^−^.

Structural parameters of the average local Fe neighborhood (type and the average number of neighbors, the radii, and Debye–Waller factor of neighbor shells) are quantitatively resolved from the Fe EXAFS spectra by comparing the measured EXAFS signal with model signal, constructed ab initio with the FEFF6 program code [50], in which the photoelectron scattering paths are calculated ab initio from a tentative spatial distribution of Fe neighbor atoms. The atomic species of neighbors are identified in the fit by their specific scattering factor and phase shift. The FEFF model of Fe cations in the solution was based on previous structural studies of FeCl_2_ (aq), which indicate that different Fe complexes ([FeCl_x_(H_2_O)_6−x_]^2−x^ for x = 0–2) with octahedral geometry may be expected in FeCl_2_ (aq) at concentrations up to 4 molL^−1^ and temperatures below 100 °C [31,32,33,49]. The model structures of Fe-ionic species in FeCl_2_ (aq) are schematically presented in Figure 5.

The FEFF model of the nearest Fe coordination shell included two single scattering paths, one for Cl neighbors at 2.3 Å and the second for oxygen neighbors at 2.1 Å, and all multiple (triangular and linear) scattering paths with a total length up to 5 Å. Three variable parameters for each type of neighbor in the first coordination sphere were varied: the coordination number (N), the distance to the neighbor atoms (R), and the Debye–Waller factors (σ^2^). The amplitude reduction factor (*S*_0_^2^) was fixed at the value of 0.85. A shift of energy origin Δ*E*_o_, common to all scattering paths, was also varied. The total number of O and Cl neighbor atoms in the nearest Fe coordination shell was constrained to six and arranged in a distorted octahedron, as suggested by XANES analysis. The parameters of multiple scattering paths within the distorted octahedra of nearest neighbors were constrained with the values of structural parameters obtained by modeling the single scattering paths on the same neighbors.

The spectra were fitted in the *k* range from 3.4 Å^−1^ to 13 Å^−1^. A very good fit is obtained for all spectra in the R range of 1 Å up to 5 Å. (Figure 2, Figure 3 and Figure 4). A complete list of the best-fit parameters is listed in Table 1. The parameters of multiple scattering paths are listed in Table S1 in the Supplementary Materials.

In all cases, oxygen and chlorine atoms were identified in the nearest Fe coordination shell, with the Fe–O distance of 2.095 ± 0.005 Å and Fe–Cl distance of 2.33 ± 0.02 Å, in agreement with previous findings [31,32,33]. In the 1 molL^−1^ FeCl_2_ (aq) at RT, we found 5.0 oxygen and 1.0 chlorine neighbors on average, clearly indicating that the dominant species are [FeCl(H_2_O)_5_]^+^ complexes. A systematic increase in the number of Cl neighbors and a decrease in the number of O neighbors is observed at higher temperatures (Table 1).

The Debye–Waller factors of Fe-Cl coordination are significantly larger, indicating larger static disorder for Cl neighbors present in the first hydration shell of the octahedral Fe^2+^ ion. A smaller increase of the Debye–Waller factor of both species is detected with increased temperature as expected due to larger thermal disorder in the first coordination shell at higher temperatures. The results indicate that at higher temperatures the [FeCl_2_(H_2_O)_4_]^0^ complexes predominate over [FeCl(H_2_O)_5_]^+^.

Similar results are obtained also for solutions with higher concentrations at RT (Table 1). A systematic increase in the number of Cl neighbors and a decrease in the number of O neighbors is observed at higher concentrations, indicating that for the 3 molL^−1^ and 4 molL^−1^ solutions [FeCl_2_(H_2_O)_4_]^0^ are dominant complexes already at RT.

The EXAFS results indicate similar local structure around Fe^2+^ cations in FeCl_2_ (aq) as observed for Zn^2+^ cations in zinc(II) chloride in aqueous solution [44]. For a concentration of 1 molL^−1^, the existence of a six-fold Zn coordination with about 4.8 water molecules and 1.2 Cl^−^ ions were determined. With an increase in the Cl^−^ concentration, the water molecules bound to the Zn^2+^ ion are gradually exchanged with the chloride ions.

## 3. Discussion

The increase in the population of Cl^−^ ions in the first coordination sphere of the Fe-ionic species with temperature and concentration can be rationalized based on the obtained Fe K-edge EXAFS results (Table 2). At the low temperature and low FeCl_2_ concentration, the Fe^2+^ ions in the FeCl_2_ (aq) are octahedrally coordinated with one Cl^−^ and five H_2_O, which can be written as [FeCl(H_2_O)_5_]^+^. The polar water molecules are oriented with their negative oxygen side towards Fe^2+^ and all five Fe-O distances are the same (within error bars)—2.095 ± 0.005 Å (Figure 5). The Cl^−^ ion is bonded to Fe at a distance of 2.33 ± 0.02 Å, which is a characteristic Fe-Cl bond distance for chloride ions in the first hydration shell of the octahedral Fe^2+^ ion [33]. The formation of intramolecular hydrogen bonds between the hydrogen atoms of the basal water molecules and the highly electronegative apical chlorine can be expected in such a tightly bound hydration complex. This restrains the orientation of the water molecules within the complex ionic structure and does not allow for a significant dynamic structure disorder as noticed by Roscioni et al. [46] for the hydration shell around Zn^2+^. In addition, the electronegativity of chlorine shifts the electron density away from the apical oxygen, which results in the weakening and destabilizing of its electrostatic bond with Fe. As a consequence, with an increase in temperature, the breaking of the bond becomes more probable, and the substitution of the apical water molecule by the Cl^−^ ion, which due to its charge can form a stronger electrostatic bond, possible (Figure 5).

The values of the Fe-O Debye–Waller factors of about 0.007 Å^2^ are typical for the hydration shell of divalent ions in solutions [31,33], where the metal cation forms a quite stable hydration complex and fluctuations of the first-shell water molecules are due to the thermal motion only [46]. The Debye–Waller factors of Fe-Cl coordination are significantly larger, indicating larger static disorder for the Cl neighbors present in the first hydration shell of the octahedral Fe^2+^ ionic complex.

The small increase in the Debye–Waller factors with temperature and concentration indicates that the Fe^2+^ cation forms a quite stable and tightly bound hydration complex. This again supports the presence of the intramolecular hydrogen bonds between the water molecules and two apical chlorine ions, which restrain the thermal oscillations in the high-temperature Fe[Cl_2_(H_2_O)_4_]^0^ even more than in [FeCl(H_2_O)_5_]^+^.

Apparently, the described substitution mechanism decreases the entropy of the system because the initially free Cl^−^ ions get bound to Fe^2+^ to form the neutral Fe[Cl_2_(H_2_O)_4_]^0^ complex. This is why the system at low temperature and low chloride concentration prefers one and not two Cl^−^ in the first coordination shell. However, with the increase in temperature, the increased thermal oscillation of the apical water molecule further destabilizes its bond with Fe. The decrease in the substitutional enthalpy overcomes the entropy drop (-T∆S) and makes the change of free energy for this process negative, thus enabling the substitution.

The observed change in the Fe coordination shell with the increase in the FeCl_2_ concentration can also be explained considering the thermodynamic state of the system. Here, we have to emphasize that we are working with highly concentrated solutions up to 4 molL^−1^. In such solution, the nominal species ratio Fe^2+^:Cl^−^:H_2_O is about 1:2:14. Taking into account the structure of the monovalent ion complex, the ratio [FeCl(H_2_O)_5_]^+^:Cl^−^:H_2_O becomes 1:1:9. This causes the shortage of the water molecules available for hydration of the Cl^−^ ions. The studies show that a typical first hydration sphere of Cl^−^ ion consists of about 6 water molecules [51,52]. Not accounting for the outer hydration spheres, this makes the species ratio [FeCl(H_2_O)_5_]^+^:[Cl(H_2_O)_6_]^−^:H_2_O to only be 1:1:3. So, the majority of the water molecules are used up for the first hydration sphere of the present ionic species, and not enough are available for outer hydration layers. This is thermodynamically very unfavorable. The free energy of such a system highly increases and destabilizes the system. As a consequence, the formation of the neutral Fe[Cl_2_(H_2_O)_4_]^0^ species becomes thermodynamically favorable. For each transformation of [FeCl(H_2_O)_5_]^+^ into Fe[Cl_2_(H_2_O)_4_]^0^, one water molecule is released in addition to the water molecules that are released from the Cl^−^ hydration sphere. 

**Figure 6:**
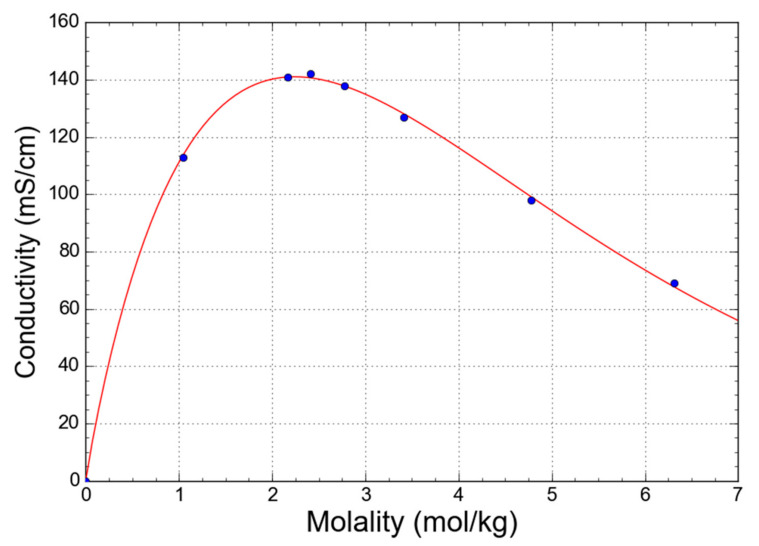
The conductivity of FeCl_2_ (aq) as a function of concentration (blue dots) and the corresponding fit (red line) of the conductivity model developed by Zhang et al. [48].

With the increase in concentration, the association of the charged Cl^−^ and [FeCl(H_2_O)_5_]^+^ into neutral Fe[Cl_2_(H_2_O)_4_]^0^ must influence the conductivity of the solutions due to the decrease in the concentration of the charged ionic species. Figure 6 shows that the conductivity in the dilute regime increases with the concentration. This is expected as the ionic mobility and the population of species are not affected much by the increase in the concentration whereas the number of charge carriers (i.e., charged ionic species) significantly increases. With a further increase in the concentration, the ionic mobility is getting hindered due to ionic interactions, and the population of the ionic species changes in favor of the neutral species. This makes the initial increase in conductivity to slow down and reaches the maximum value at around 2.2 mol·kg^−1^. Eventually, at even higher concentrations, the conductivity decreases sharply as a result of an ever lower concentration of charged ionic species and a hindered ionic mobility due to a lack of free water molecules. The decrease is much sharper than in the case of, e.g., divalent CaCl_2_. Up to the concentration of 4.5 mol·kg^−1^, the conductivity of CaCl_2_ (aq) decreases from its maximum for only about 10%, while it decreases by 35% for FeCl_2_ (aq). 

The observed association of the ionic species that causes the nonlinear relationship between a concentration of the electrolyte and a concentration of free ions can also be deduced from the model developed by Zhang et al. [48]:

(1) $$\kappa =\left(P_{1}T+P_{2}\right)m^{n}\exp\left(-\frac{P_{3}m}{T-P_{4}}\right)$$

where *κ*, *T*, and *m* are conductivity, temperature, and molality. *P*_1_, *P*_2_, *P*_3_, *P*_4_, and *n* are constants independent of concentration and temperature.

The measurements from Figure 6 were fitted by Equation (1) to extract the model parameters. If the exponential value *n* is equal to 1, the relationship is linear while the deviation from 1 reflects nonlinearity. For NaCl (aq) and CaCl_2_ (aq), the *n* value is 1 ± 0.005, which indicates the negligible association of ions. For FeCl_2_ (aq) the *n* value is 0.94, which supports the conclusions of XAS studies on the ionic association. 

## 4. Materials and Methods

### 4.1. Sample Preparation

The FeCl_2_ (aq) solutions with concentrations from 1 molL^−1^ to 4 molL^−1^ were prepared from FeCl_2_·4H_2_O (Acros Organics 70 Mesh, <212 µm particle size, 99+% purity) and followed by a reduction procedure to eliminate traces of Fe (III). The reduction procedure consisted of adding 5 mL of HCl per 1 L of solution and Fe powder in excess. Typically, a clear green-colored solution with pH from 3.5 to 4 is obtained in 12–36 h at room temperature. When the target pH is reached the excess of iron was removed. The sample solutions were stored under reductive conditions in air-tight transparent borosilicate glass bottles under a nitrogen gas atmosphere at room temperature during transportation. The correct FeCl_2_ (aq) concentration was recalculated to account for the reduction procedure.

The measurements of pH, conductivity, and temperature were performed using Phoenix EC45-Multi probes. The conductivity measuring cell, Sentek VPT80C10, was calibrated using Hamilton ZDL 300 mL standard solution with a conductivity of 100 mScm^−1^ at 25 °C. De-ionized water (Grade 2) was used for all the prepared solutions. 

### 4.2. In Situ XAS of FeCl_2_ (aq) As a Function of Concentration and Temperature

**Figure 7:**
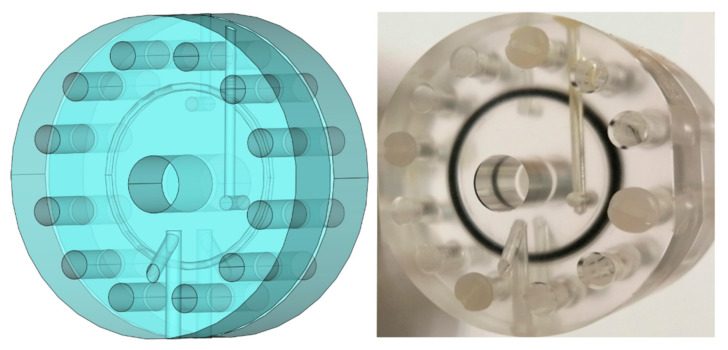
CAD drawing and photograph of the experimental PMMA liquid absorption cell, designed and manufactured on-purpose for the reported XAS measurements.

In situ Fe K-edge X-ray absorption near edge structure (XANES) and extended X-ray absorption fine structure (EXAFS) measurements of 1 molL^−1^ FeCl_2_ aqueous solution in a temperature range from RT to 80 °C and the FeCl_2_ aqueous solutions at higher concentrations (2, 3, and 4 molL^−1^) at RT were performed at P65 beamline of PETRA III, at DESY in Hamburg. In addition, 1 molL^−1^ FeCl_3_ aqueous solution and crystalline FeSO_4_·7H_2_O were measured at room temperature for comparison. The XAS spectra were measured in transmission detection mode. We used our on-purpose designed experimental setup which consists of a PMMA (Polymethyl methacrylate) liquid absorption cell (Figure 7) mounted inside a remotely controlled heating system. The tubular oven allowed the in-situ temperature-controlled measurements at well-defined 10 °C temperature steps. The PMMA cell windows were machined down to 0.2 mm thickness to minimize the absorption. The optimal absorption thickness of the sample solution was adjusted over EPDM rubber spacers thickness providing a solution layer from 0.1 to 0.3 mm. The solution was inserted in the liquid absorption cell by a syringe, in a glove box under a protective atmosphere to prevent oxidation of the Fe and sealed. The sealed cell was inserted in the tubular oven, which was mounted on the beamline between the first and second ionization cells. The oven was closed with aluminum foil (20 µm) windows and filled with He protective atmosphere. The absorption cell was equipped with a thermocouple to read the temperature of the liquid during the in situ XAS experiments.

A Si(111) double crystal monochromator was used with an energy resolution of about 1 eV at 7 keV. The beam size on the sample was 1.5 mm horizontally and 0.2 mm vertically. The absorption spectra were measured in the energy region from −150 eV to +1000 eV relative to the Fe K-edge (7112 eV) in continuous fast (3 min) scans and re-binned to equidistant energy steps of 0.25 eV in the XANES region and equidistant k steps of 0.05 Å^−1^ in the EXAFS region. The exact energy calibration was established with simultaneous absorption measurement on a 5-micron thick Fe foil placed between the second and third ionization detector. The Fe K-edge XAFS spectra were measured first at RT and then during heating at well-defined 10 °C temperature steps up to 80 °C. At each intermediate temperature state, we performed three to five repetitions of the same scan to check the reproducibility and to improve the signal-to-noise ratio. We measured XAS scans on different spots of the absorption cell window to reduce the eventual effects of radiation damage.

The quantitative analysis of XANES and EXAFS spectra was performed with the Demeter (IFEFFIT) program package [42] in combination with the FEFF6 program code for ab initio calculation of photoelectron scattering paths [50].

## Figures and Tables

**Table 1:** Parameters of the nearest coordination shells around Fe cations in the FeCl_2_ samples: average number of neighbor atoms (N), distance (R), and Debye–Waller factor (σ^2^). Uncertainty of the last digit is given in parentheses. The best fit is obtained with a total number of O and Cl neighbors constrained to 6, the amplitude reduction factor *S*_0_^2^ = 0.85, and the shift of the energy origin Δ*E*_o_ = −4 ± 1 eV. The R-factor (quality of fit parameter) is listed in the last column. Uncertainty of the last digit is given in parentheses.

Fe Neighbors	N	R [Å]	σ^2^ [Å^2^]	R-Factor
**1 molL^−1^ FeCl_2_ aq. solution measured at RT**				
O	5.0(3)	2.095(5)	0.0072(5)	0.00030
Cl	1.0	2.31(2)	0.015(3)	
**1 molL^−1^ FeCl_2_ aq. solution measured at T = 30 °C**				
O	4.9(3)	2.096(5)	0.0071(5)	0.00020
Cl	1.1	2.34(2)	0.015(3)	
**1 molL^−1^ FeCl_2_ aq. solution measured at T = 40 °C**				
O	4.8(3)	2.093(5)	0.0072(5)	0.00016
Cl	1.2	2.33(2)	0.015(3)	
**1 molL^−1^ FeCl_2_ aq. solution measured at T = 50 °C**				
O	4.6(3)	2.095(5)	0.0073(5)	0.00013
Cl	1.4	2.33(2)	0.017(3)	
**1 molL^−1^ FeCl_2_ aq. solution measured at T = 60 °C**				
O	4.5(3)	2.095(5)	0.0075(5)	0.00009
Cl	1.5	2.33(2)	0.018(3)	
**1 molL^−1^ FeCl_2_ aq. solution measured at T = 80 °C**				
O	4.5(3)	2.094(5)	0.0082(5)	0.00012
Cl	1.5	2.33(2)	0.017(3)	
**2 molL^−1^ FeCl_2_ aq. solution measured at RT**				
O	4.5(3)	2.103(5)	0.0065(5)	0.00015
Cl	1.5	2.35(2)	0.021(3)	
**3 molL^−1^ FeCl_2_ aq. solution measured at RT**				
O	4.0(3)	2.097(5)	0.0066(5)	0.00012
Cl	2.0	2.37(2)	0.019(3)	
**4 molL^−1^ FeCl_2_ aq. solution measured at RT**				
O	4.0(3)	2.097(5)	0.0066(5)	0.00012
Cl	2.0	2.37(2)	0.019(3)	

**Table 2:** Number of O and Cl neighbors in the first coordination shell around Fe^2+^ cations in the selected temperature and concentration range, as determined by EXAFS analysis. The average Fe-O and Fe-Cl distances are 2.095 ± 0.005 Å and 2.33 ± 0.02 Å, respectively. Uncertainty in the coordination numbers is ±0.3. The total number of neighbors in the first coordination shell in the EXAFS fit was fixed to 6.

Temperature (°C)	Average Number of Neighbors in 1 molL^−1^ FeCl_2_		FeCl_2_ (aq) C. (molL^−1^)	Average Number of Neighbors at RT	
	Cl	O		Cl	O
20	1.0	5.0	1	1.0	5.0
30	1.1	4.9	2	1.5	4.5
40	1.2	4.8	3	2.0	4.0
50	1.4	4.6	4	2.0	4.0
60	1.5	4.5			
80	1.5	4.5			

## Supplementary Materials

The following supporting information can be downloaded online, Table S1: Parameters of photoelectron multiple scattering paths on the neighbors in the nearest coordination shells around Fe cations in the FeCl_2_ samples.
