# Supplementary material for: Structure and Population of Complex Ionic Species in FeCl2 Aqueous Solution by X-ray Absorption Spectroscopy
Source: Molecules. 2022 Jan 19;27(3):642. doi: 10.3390/molecules27030642 (PMC8839570; doi:10.3390/molecules27030642)
Supplement: Supplementary file 1 [file molecules-27-00642-s001.zip › molecules-1535557-SI.pdf]

Article

Structure and Population of Complex Ionic Species in $FeCl_2$ Aqueous Solution by X-Ray Absorption Spectroscopy

Uroš Luin [1], Iztok Arčon [1,2] and Matjaz Valant [1,*]

Table S1. Parameters of photoelectron multiple scattering paths on the neighbours in the nearest coordination shells around Fe cations in the $FeCl_2$ samples. All multiple (triangular and linear) scattering paths with a total length up to 5 Å in the two in $FeCl_2$ (aq) species ($[FeCl(H_2O)_5]^+$ and $[FeCl_2(H_2O)_4]$ schematically presented in Figure 2.) were included in the FEFF model. Following parameters are listed in the table: path degeneracy (N), relative amount of each path (N_{rel}), based on relative amount of of the two $FeCl_2$ (aq) species (uncertainty ±0.3) , half-path length (R), and Debye-Waller factor of each path (σ^2). Uncertainty of the last digit is given in parentheses. The parameters of multiple scattering paths within the distorted octahedra of nearest neighbors were constrained with the values of structural parameters obtained by modeling the single scattering paths on the same neighbors, listed in Table 1. The best fit is obtained with a total number of O and Cl neighbors constrained to 6, the amplitude reduction factor S_0^2=0.85, and the shift of the energy origin ΔE_o = -4 ±1 eV.

MS paths	N* N_{rel}	R [Å]	σ^2 [Å^2]
1 molL^{-1} $FeCl_2$ aq. solution measured at RT			
Fe-O-O-Fe	16*1.0	3.63(1)	0.011(1)
Fe-O-Cl-Fe	16*0.1	3.80(3)	0.026 (6)
Fe-O-O-Fe	4*1.0	4.20(1)	0.011(1)
Fe-O-Fe-O-Fe	4*1.0	4.20(1)	0.008(1)
Fe-O-Fe-O-Fe	5*1.0	4.20(1)	0.008(1)
Fe-Cl-Cl-Fe	2*0.0	4.62(4)	0.015(3)
Fe-Cl-Fe-Cl-Fe	2*0.0	4.62(4)	0.015(3)

MS paths	N* Nrel	R [Å]	σ^2 [Å2]
1 molL^{-1} $FeCl_2$ aq. solution measured at T = 30 °C			
Fe-O-O-Fe	16*0.9	3.63(1)	0.011(1)
Fe-O-Cl-Fe	16*0.2	3.83(3)	0.026 (6)
Fe-O-O-Fe	4*1.0	4.20(1)	0.011(1)
Fe-O-Fe-O-Fe	4*1.0	4.20(1)	0.008(1)
Fe-O-Fe-O-Fe	5*1.0	4.20(1)	0.008(1)
Fe-Cl-Cl-Fe	2*0.1	4.68(4)	0.015(3)
Fe-Cl-Fe-Cl-Fe	2*0.1	4.68(4)	0.015(3)

MS paths	N* N_{rel}	R [Å]	σ^2 [Å^2]
1 molL^{-1} $FeCl_2$ aq. solution measured at T = 40 °C			
Fe-O-O-Fe	16*0.9	3.63(1)	0.011(1)
Fe-O-Cl-Fe	16*0.3	3.82(3)	0.026 (6)

Fe-O-O-Fe	4*1.0	4.20(1)	0.011(1)
Fe-O-Fe-O-Fe	4*1.0	4.20(1)	0.008(1)
Fe-O-Fe-O-Fe	5*1.0	4.20(1)	0.008(1)
Fe-Cl-Cl-Fe	2*0.2	4.68(4)	0.015(3)
Fe-Cl-Fe-Cl-Fe	2*0.2	4.68(4)	0.015(3)

MS paths	N* Nrel	R [Å]	σ^2 [Å2]
1 molL^{-1} $FeCl_2$ aq. solution measured at T = 50 °C			
Fe-O-O-Fe	16*0.8	3.63(1)	0.011(1)
Fe-O-Cl-Fe	16*0.5	3.82(3)	0.028 (6)
Fe-O-O-Fe	4*1.0	4.20(1)	0.011(1)
Fe-O-Fe-O-Fe	4*1.0	4.20(1)	0.008(1)
Fe-O-Fe-O-Fe	5*0.9	4.20(1)	0.008(1)
Fe-Cl-Cl-Fe	2*0.4	4.68(4)	0.017(3)
Fe-Cl-Fe-Cl-Fe	2*0.4	4.68(4)	0.017(3)
MS paths	N* N_{rel}	R [Å]	σ^2 [Å^2]
1 molL^{-1} $FeCl_2$ aq. solution measured at T = 60 °C			
Fe-O-O-Fe	16*0.9	3.63(1)	0.012(1)
Fe-O-Cl-Fe	16*0.6	3.82(3)	0.029 (6)
Fe-O-O-Fe	4*1.0	4.20(1)	0.012(1)
Fe-O-Fe-O-Fe	4*1.0	4.20(1)	0.008(1)
Fe-O-Fe-O-Fe	5*0.9	4.20(1)	0.008(1)
Fe-Cl-Cl-Fe	2*0.5	4.68(4)	0.018(3)
Fe-Cl-Fe-Cl-Fe	2*0.5	4.68(4)	0.018(3)

MS paths	N* Nrel	R [Å]	σ^2 [Å2]
1 molL^{-1} $FeCl_2$ aq. solution measured at T = 80 °C			
Fe-O-O-Fe	16*0.8	3.63(1)	0.012(1)
Fe-O-Cl-Fe	16*0.6	3.82(3)	0.029 (6)
Fe-O-O-Fe	4*1.0	4.20(1)	0.011(1)
Fe-O-Fe-O-Fe	4*1.0	4.20(1)	0.008(1)
Fe-O-Fe-O-Fe	5*0.9	4.20(1)	0.008(1)
Fe-Cl-Cl-Fe	2*0.5	4.68(4)	0.017(3)
Fe-Cl-Fe-Cl-Fe	2*0.5	4.68(4)	0.017(3)
MS paths	N* N_{rel}	R [Å]	σ^2 [Å^2]
2 molL^{-1} $FeCl_2$ aq. solution measured at RT			
Fe-O-O-Fe	16*0.8	3.63(1)	0.009(1)
Fe-O-Cl-Fe	16*0.6	3.83(3)	0.029 (6)
Fe-O-O-Fe	4*1.0	4.21(1)	0.009(1)
Fe-O-Fe-O-Fe	4*1.0	4.21(1)	0.007(1)
Fe-O-Fe-O-Fe	5*0.9	4.21(1)	0.007(1)
Fe-Cl-Cl-Fe	2*0.5	4.71(4)	0.021(3)
Fe-Cl-Fe-Cl-Fe	2*0.5	4.71(4)	0.021(3)

MS paths	N* N_{rel}	R [Å]	σ^2 [Å^2]
3 molL^{-1} $FeCl_2$ aq. solution measured at RT			

Fe-O-O-Fe	16*0.5	3.63(1)	0.009(1)
Fe-O-Cl-Fe	16*1.0	3.86(3)	0.030(6)
Fe-O-O-Fe	4*1.0	4.20(1)	0.009(1)
Fe-O-Fe-O-Fe	4*1.0	4.20(1)	0.007(1)
Fe-O-Fe-O-Fe	5*0.8	4.20(1)	0.007(1)
Fe-Cl-Cl-Fe	2*1.0	4.75(4)	0.019(3)
Fe-Cl-Fe-Cl-Fe	2*1.0	4.75(4)	0.019(3)

MS paths	N* Nrel	R [Å]	σ2 [Å2]
4 $molL^{-1}$ $FeCl_2$ aq. solution measured at RT			
Fe-O-O-Fe	16*0.5	3.63(1)	0.009(1)
Fe-O-Cl-Fe	16*1.0	3.86(3)	0.030(6)
Fe-O-O-Fe	4*1.0	4.20(1)	0.009(1)
Fe-O-Fe-O-Fe	4*1.0	4.20(1)	0.007(1)
Fe-O-Fe-O-Fe	5*0.8	4.20(1)	0.007(1)
Fe-Cl-Cl-Fe	2*1.0	4.75(4)	0.019(3)
Fe-Cl-Fe-Cl-Fe	2*1.0	4.75(4)	0.019(3)
